# Increase in invasive group A streptococcal infections and emergence of novel, rapidly expanding sub-lineage of the virulent *Streptococcus pyogenes* M1 clone, Denmark, 2023

**DOI:** 10.2807/1560-7917.ES.2023.28.26.2300291

**Published:** 2023-06-29

**Authors:** Thor Bech Johannesen, Charlotte Munkstrup, Sofie Marie Edslev, Sharmin Baig, Stine Nielsen, Tjede Funk, Dennis Karsten Kristensen, Lars Hervig Jacobsen, Signe Fischer Ravn, Niels Bindslev, Sophie Gubbels, Marianne Voldstedlund, Pikka Jokelainen, Søren Hallstrøm, Astrid Rasmussen, Karl Gústaf Kristinsson, David Fuglsang-Damgaard, Ram B Dessau, Agnieszka Barbara Olsén, Christian Salgaard Jensen, Annette Skovby, Svend Ellermann-Eriksen, Thøger Gorm Jensen, Esad Dzajic, Claus Østergaard, Steen Lomborg Andersen, Steen Hoffmann, Peter Henrik Andersen, Marc Stegger

**Affiliations:** 1 Bacteria, Parasites and Fungi, Statens Serum Institut, Copenhagen, Denmark; 2 Infectious Disease Epidemiology and Prevention, Statens Serum Institut, Copenhagen, Denmark; 3 Data Integration and Analysis, Statens Serum Institut, Copenhagen, Denmark; 4 Infectious Disease Preparedness, Statens Serum Institut, Copenhagen, Denmark; 5 Department of Clinical Microbiology, Landspitali – the National University Hospital, Reykjavik, Iceland; 6 Faculty of Medicine, University of Iceland, Reykjavik, Iceland; 7 Department of Clinical Microbiology, Aalborg University Hospital, Aalborg, Denmark; 8 Department of Clinical Microbiology, Zealand University Hospital, Slagelse, Denmark; 9 Department of Regional Health Research, University of Southern Denmark, Odense, Denmark; 10 Department of Clinical Microbiology, Herlev and Gentofte Hospital – University Hospital, Herlev, Denmark; 11 Department of Clinical Microbiology, Rigshospitalet, Copenhagen, Denmark; 12 Copenhagen University Hospital – Amager and Hvidovre, Hvidovre, Denmark; 13 Department of Clinical Microbiology, Aarhus University Hospital, Aarhus, Denmark; 14 Department of Clinical Microbiology, Odense University Hospital and Research Unit of Clinical Microbiology, Odense, Denmark; 15 Clinical Diagnostic Department, Clinical Microbiology, Hospital South West Jutland, University Hospital of Southern Denmark, Esbjerg, Denmark; 16 Department of Clinical Microbiology, Lillebælt Hospital, University Hospital of Southern Denmark, Vejle, Denmark; 17 Department of Clinical Microbiology, Sønderjylland Hospital, University Hospital of Southern Denmark, Aabenraa, Denmark; 18 Antimicrobial Resistance and Infectious Diseases Laboratory, Harry Butler Institute, Murdoch University, Perth, Australia

**Keywords:** *Streptococcus pyogenes*, group A *Streptococcus*, M1, M1DK, virulence, bacterial genomics, Denmark, Iceland

## Abstract

A highly virulent sub-lineage of the *Streptococcus pyogenes* M1 clone has been rapidly expanding throughout Denmark since late 2022 and now accounts for 30% of the new invasive group A streptococcal infections. We aimed to investigate whether a shift in variant composition can account for the high incidence rates observed over winter 2022/23, or if these are better explained by the impact of COVID-19-related restrictions on population immunity and carriage of group A *Streptococcus*.

 An increase in incidence rates of invasive (iGAS) and non-invasive (nGAS) group A *Streptococcus* infection has been reported by several countries across Europe during the 2022/23 winter season [1-3]. Through analysis of all whole genome sequencing (WGS) data acquired for national surveillance of iGAS in Denmark since 2018, we aimed to investigate current genomic developments and the impact of emerging lineages on iGAS incidence rates in 2023. In Denmark, iGAS is not notifiable except in case of meningitis, however, test results from all 10 Departments of Clinical Microbiology (DCMs) are submitted to the Danish Microbiology Database (MiBa) [4] and can be used to monitor incidence rates. Iceland also experienced a higher iGAS incidence in early 2023, and we also present Icelandic WGS data on iGAS isolates from 2022 and 2023.

## Incidence of invasive group A *Streptococcus* in Denmark in 2023

We extracted all culture-positive test results for group A *Streptococcus* (GAS) since 2018 on 1 June 2023, and categorised them as iGAS, if the sample was from blood, synovial fluid, spinal fluid, peritoneal fluid, pleural fluid or usually sterile organ tissue, and as nGAS if the isolate was from any other sample type or from usually non-sterile organ tissue. Multiple positive test results from the same individual within a 30-day period were considered a single case. In total, we identified 1,265 cases of iGAS during the period January 2018 to May 2023 across Denmark (2023 population 5.9 million [5]).

**Figure 1:**
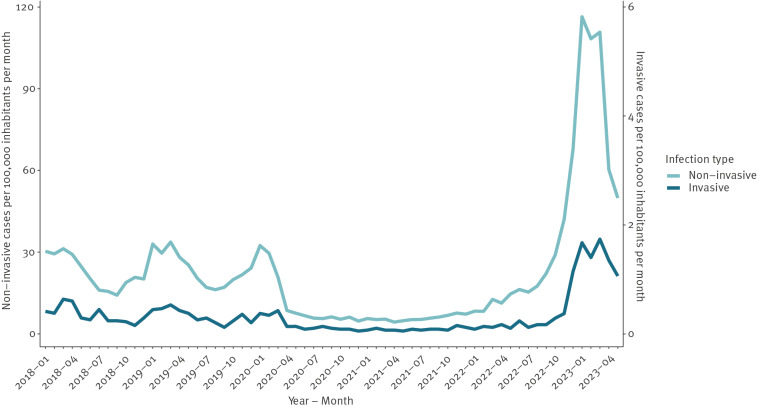
Number of non-invasive (n = 86,793) and invasive (n = 1,265) laboratory-diagnosed infections of group A *Streptococcus* per 100,000 inhabitants per month, Denmark, January 2018–May 2023

Denmark experienced historically low iGAS incidence rates during COVID-19-related restrictions, which ended in February 2022. Case numbers began to increase rapidly in November 2022, peaking in January 2023 with a monthly GAS incidence of 118 per 100,000 inhabitants, i.e. 3.5 times the peak rates seen in 2018/19, and a monthly iGAS incidence rate of 1.7 per 100,000, thus 3 times the peak rates seen in 2018/19 (Figure 1).

People 85 years or older had the highest iGAS incidence rates, peaking at 7.4 per 100,000 in the age group per month, but the highest relative increase compared with pre-COVID-19 restrictions was observed among children younger than 5 years, which peaked at 3.2 per 100,000 in the age group in March 2023. Mortality rates were similar to previous years across all age groups: 30% among people 85 years or older and less than 5% among children under 5 years. There was no substantial difference in prevalence between sexes during the winter season, with females accounting for 47.5% (222/467) of the overall cases, 41.7% (15/36) among children younger than 5 years, 52.8% (105/199) in ages 5-64 and 44.0% (102/232) among people aged 65 years and older.

The exact testing frequency was unknown but estimated from limited local data to be higher than usual in winter 2022/23, and comparisons of nGAS incidence rates between years were therefore less robust. Assuming that the testing frequency remained constant, a 2.5–4.5-fold increase in nGAS incidence was observed across all age groups relative to 2018/19. The highest increase, as well as the highest overall incidence rate, was observed among children younger than 5 years, and elevated incidence rates were seen throughout all regions of Denmark.

In Iceland, 46 cases of iGAS have been reported in 2023 as of May 7, compared to an annual average of 15 cases in the period 2010 to 2019, with a particularly noticeable increase among young children. In 2022 and 2023, children younger than 5 years accounted for 17.4% (12/69) of the cases versus 10.8% (47/436) in the period 1975 to 2021. People aged 60 years and older accounted for 33.3% (23/69) of the cases, a noticeably lower proportion than 49.3% (214/434) in the period 1975 to 2022. Like in Denmark, iGAS prevalence in males and females was similar in all age groups (data not shown).

## Molecular identification of a novel M1 sub-lineage

**Figure 2:**
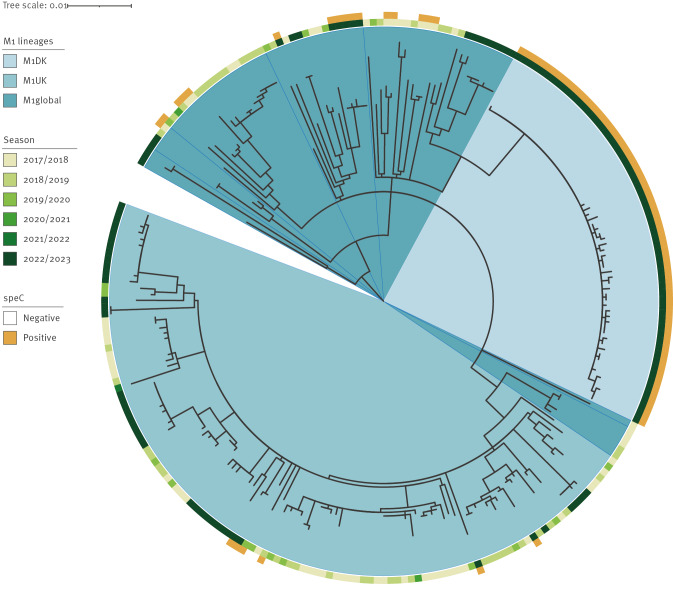
Core genome phylogeny of all *Streptococcus pyogenes* M1 isolates sampled from invasive infections with available whole genome sequencing data, Denmark, January 2018–February 2023 (n = 251)

**Figure 3:**
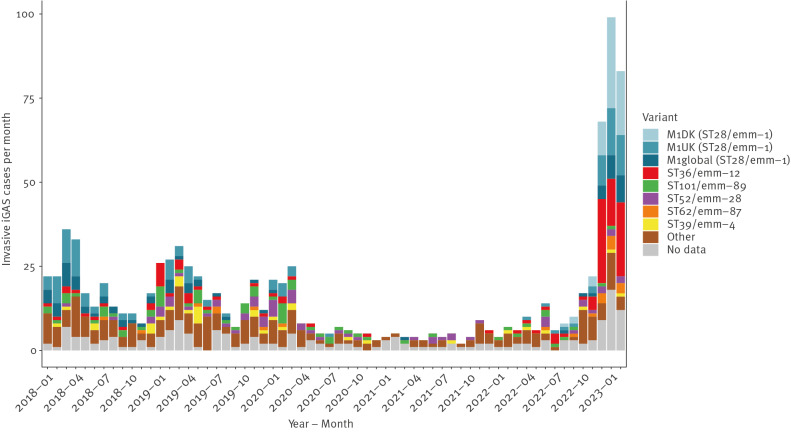
Distribution of variants in laboratory-diagnosed invasive group A streptococcal infections, Denmark, January 2018–February 2023 (n = 1,019)

DK: Denmark; EMM: the M protein; iGAS: invasive group A *Streptococcus;* ST: sequence type; UK: UK.

High-quality whole genome sequencing data (Illumina) was available for 839 (82%) of 1,019 iGAS cases identified in the period January 2018 to February 2023. The M1 clone (ST28/EMM-1.0), along with ST36/EMM-12.0, were the most common causes of iGAS during the recent increase, accounting for 87 (57%) and 36 (24%) cases, respectively, in the peak months of January and February 2023. In Denmark, M1 has been the leading cause of iGAS, but phylogenetic (Figure 2) and accessory genome analyses using long-read sequencing data (Oxford Nanopore Technologies) [6] on selected isolates, revealed noteworthy trends and developments within the M1 clone. Like in many other countries, the M1_UK_ lineage, first observed in 2010 [7], had become the dominant cause of iGAS in Denmark before the implementation of COVID-19-related restrictions. The recent surge in cases has, however, coincided with the rise of a novel lineage (M1_DK_), which was first observed in August 2022, and accounted for 30% of all sequenced iGAS isolates in 2023 (Figure 3). M1_DK_ was highly prevalent in all regions of Denmark and across all ages. It accounted for 25–39% of iGAS cases in each age group, making it the most common variant of iGAS among all age groups except for 15–44 year-olds, where ST36 was more common. M1_DK_ does not possess any mutations previously shown to increase expression of exotoxin *speA* in the M1 clone [7,8], nor any mutations characterising the M1_UK_ lineage [7]. In addition to constituting a distinct, rapidly expanding phylogenetic clade, the M1_DK_ lineage is characterised by the acquisition of a bacteriophage containing the exotoxin *speC*, which is absent from the ancestral M1_global_ and M1_UK_ strains. Phylogenetic analysis of Danish iGAS isolates between 2018 and 2023 revealed multiple instances of *speC*-acquisition by circulating M1_UK_ and M1_global_ strains, but none of these lineages had previously led to a substantial number of invasive cases. Further information on genomic characteristics of M1_DK_ and the bacteriophage, including genomic reference strains, can be found in the Supplement.

Clades corresponding to the M1_DK_ and M1_UK_ lineages are indicated in lighter shades of blue. Sampling time is indicated in green shades at tips, with darker green indicating more recent samples. Seasons are defined starting 1 July and ending 30 June. Isolates positive for *speC* are marked with yellow.

The most prevalent sequence types are listed, and M1 (ST28/EMM-1.0) isolates have been further subtyped into three lineages. Isolates marked ‘No data’ are from cases without whole genome sequencing data. Variant type is determined by in silico multilocus sequence typing and core genome single nt polymorphism analysis.

To categorise the genomics of iGAS in Iceland, WGS data from 43 isolates sampled from patients hospitalised with serious disease in 2022 and 2023 were analysed. Unlike in Denmark, the M1_UK_ lineage remained the dominant cause of iGAS in Iceland, accounting for 19 (44%) cases. A single case was caused by the M1_global_ lineage, while M1_DK_ was not observed.

## Risk of invasive infection

We collected information on deaths from the Danish Civil Registration System and extracted information on intensive care treatment and duration of hospital admissions from the Danish National Patient Registry [9] for iGAS cases since 2018. The mortality rate and risk of requiring intensive care treatment of each variant were compared with all other variants combined using Fisher’s exact test, while duration of hospital admission was compared using Mann–Whitney U test (R statistical software v4.0.2) [10]. The mortality rate (Table 1) and length of hospital admission for iGAS infections were similar for all variants, but patients infected with M1 variants more often required semi-intensive or intensive care treatment (Table 1).

**Table 1:** Variants of invasive group A *Streptococcus* isolates from cases treated in semi-intensive or intensive care units and from fatal cases, Denmark, January 2018–February 2023 (n = 839)

Variant	n	Cases in intensive care				Fatal cases			
		n	%	p value	OR (95% CI)	n	%	p value	OR (95% CI)
M1_DK_ (ST28/EMM-1)	62	24	38.7	0.002	2.38 (1.32–4.2)	10	16.1	0.9	1.08 (0.48–2.24)
M1_UK_ (ST28/EMM-1)	119	38	31.9	0.009	1.8 (1.14–2.8)	16	13.4	0.7	0.85 (0.45–1.52)
M1_global_ (ST28/EMM-1)	70	23	32.9	0.04	1.8 (1.01–3.13)	11	15.7	0.9	1.05 (0.48–2.09)
ST36/EMM-12	111	24	21.6	0.9	0.96 (0.56–1.58)	18	16.2	0.8	1.1 (0.6–1.92)
ST101/EMM-89	69	12	17.4	0.4	0.72 (0.34–1.39)	10	14.5	1	0.95 (0.42–1.93)
ST52/EMM-28	62	12	19.4	0.6	0.83 (0.39–1.62)	6	9.7	0.3	0.58 (0.2–1.39)
ST44/EMM-66	35	6	17.1	0.5	0.71 (0.24–1.78)	5	14.3	1	0.93 (0.28–2.49)
ST39/EMM-4	34	1	2.9	0.003	0.1 (0–0.61)	2	5.9	0.2	0.34 (0.04–1.36)
ST62/EMM-87	21	2	9.5	0.2	0.36 (0.04–1.52)	2	9.5	0.8	0.58 (0.07–2.47)
Other ST	256	45	17.6	0.03	0.66 (0.45–0.97)	47	18.4	0.09	1.41 (0.93–2.13)

CI: confidence interval; EMM: the M protein; GAS: group A *Streptococcus*; iGAS: invasive group A *Streptococcus;* nGAS: non-invasive group A *Streptococcus*; OR: Odds ratio; ST: sequence type.

p values and ORs are based on Fisher’s exact test, comparing the distribution for one variant to the combined distribution of other variants.

We compared current genomic trends in iGAS (n  =  257) and nGAS (n  =  152) isolates collected by DCMs and sampled from cases between January and February 2023. All M1 variants were more common among iGAS than among nGAS, while ST36 was overrepresented in nGAS (Table 2).

**Table 2:** Distribution of variants of group A *Streptococcus* isolates from invasive and non-invasive infections, Denmark, January–February 2023 (n = 409)

Variant	iGAS		nGAS		p value	OR (95% CI)
	n	%	n	%		
M1_DK_ (ST28/EMM-1)	46	30.3	36	14	0.0001	2.66 (1.58–4.51)
M1_UK_ (ST28/EMM-1)	26	17.1	23	8.9	0.02	2.1 (1.1–4.02)
M1_global_ (ST28/EMM-1)	15	9.9	8	3.1	0.007	3.4 (1.31–9.5)
ST36/EMM-12	36	23.7	119	46.3	0.000005	0.36 (0.22–0.57)
ST101/EMM-89	1	0.7	14	5.4	0.01	0.12 (0–0.77)
ST52/EMM-28	4	2.6	14	5.4	0.2	0.47 (0.11–1.53)
ST39/EMM-4	2	1.3	15	5.8	0.04	0.22 (0.02–0.95)
ST62/EMM-87	7	4.6	8	3.1	0.4	1.5 (0.45–4.85)
Other STs	15	9.9	20	7.8	0.5	1.3 (0.6–2.76)

EMM: the M protein; GAS: group A *Streptococcus*; iGAS: invasive group A *Streptococcus;* nGAS: non-invasive group A *Streptococcus*; ST: sequence type.

p values and odds ratios (ORs) are based on Fisher’s exact test, comparing the distribution for one variant to the combined distribution of other variants.

Although the novel M1_DK_ lineage was overrepresented in invasive cases and conveys the highest risk of requiring intensive care treatment, we estimate its virulence to be similar to the other M1 variants, which are all associated with more severe infection, and the spread of M1_DK_ has not led to a considerable increase in iGAS mortality rate or risk of intensive care. The mortality rate of iGAS in January and February 2023 was 10–20%, while 20–30% of iGAS patients received intensive care, which is similar to what was observed in the winter months of 2018/19 and 2019/20.

## Discussion

Previously, the emergence of novel variants with increased capacity for virulence has been an important factor behind iGAS incidence rates. Even single nucleotide polymorphisms or indels can significantly alter iGAS virulence [7,8,11], underlining the importance of continuous surveillance of genomic trends and identification of emerging variants [12]. A shift in distribution towards the more virulent M1 variants, and the rapid spread of the M1_DK_ lineage are both likely contributors to the high iGAS incidence in Denmark in 2023, however, these developments cannot adequately explain the surge in iGAS cases. Rather, we consider the high incidence rate of iGAS to be attributable primarily to extensive community spread. Low exposure to GAS in recent years has probably resulted in a lower level of immunity to GAS on a population level, particularly among the youngest children, a large fraction of whom had never been exposed to GAS.

It remains unclear whether the rapid expansion of M1_DK_ is driven by an inherent competitive advantage over other variants circulating in Denmark, but we consider that the expansion is primarily enabled by the unique circumstances surrounding the implementation and subsequent lifting of COVID-19-related restrictions. Lower immunity and reduced GAS transmission during lockdowns may have enhanced the rapid expansion of individual lineages. It is, however, noteworthy that M1_DK_ and ST36, the two variants with the largest increase in prevalence in 2022/23 compared with 2018/19, both carry the *speC*-gene which has been shown to facilitate nasopharyngeal colonisation in mouse models [13]. Status reports and genomic data from other countries with increased iGAS incidence, including the Netherlands [14], the UK [1,15] and Iceland (data from this study), suggest that the M1_UK_ lineage is the leading cause of iGAS in some European countries, providing further indication that the drastic increase in cases is also driven by factors beyond genomic developments.

## Conclusion

A thorough assessment of whether the surge in iGAS over winter 2022/23 was primarily driven by lowered immunity following COVID-19 related restrictions – a likely temporary circumstance, or by a higher prevalence of more virulent variants – a likely permanent change, is crucial to evaluate the risk of encountering another global increase of iGAS in coming years, and should ideally rely on whole genome sequencing data from across Europe.

## Supplementary Data

86000 Supplement
