# Supplementary material for: Increase in invasive group A streptococcal infections and emergence of novel, rapidly expanding sub-lineage of the virulent Streptococcus pyogenes M1 clone, Denmark, 2023
Source: Euro Surveill. 2023 Jun 29;28(26):2300291. doi: 10.2807/1560-7917.ES.2023.28.26.2300291 (PMC10311951; doi:10.2807/1560-7917.ES.2023.28.26.2300291)
Supplement: Supplement [file 23-00291_JOHANNESEN_SupplementaryMaterial.pdf]

This supplementary material is hosted by Eurosurveillance as supporting information alongside the article "Increased incidence of invasive group A streptococcal infections accompanied by emergence of a novel, rapidly expanding sub-lineage of the highly virulent Streptococcus pyogenes M1 variant, Denmark, 2023", on behalf of the authors, who remain responsible for the accuracy and appropriateness of the content. The same standards for ethics, copyright, attributions and permissions as for the article apply. Supplements are not edited by Eurosurveillance and the journal is not responsible for the maintenance of any links or email addresses provided therein.

Genomic characteristics of $M1_{DK}$

A complete $M1_{DK}$ genome generated from hybrid Illumina NextSeq/Nanopore minion data is available for biosample SAMEA113551919 or at https://raw.githubusercontent.com/ssi-dk/M1DK_reference_genome/main/assemblies/DK_iGAS_2022_0442.fasta

Supplementary table 1:
15 core genome SNPs which characterize $M1_{DK}$, listed by their position in the genome of reference strain MGAS50005. All listed SNPs are found in 100% of $M1_{DK}$ and none of the $M1_{UK}$ or $M1_{global}$ strains found in Denmark.

Position in MGAS50005	Gene locus	Gene	Ref	SNP
50024	M5005_Spy0031	purK	A	G
600192	M5005_Spy0596		T	C
655648	Intergenic	Intergenic	C	T
751431	M5005_Spy0750		G	A
798142	M5005_Spy0793		C	T
874083	M5005_Spy0883		G	A
890776	M5005_Spy0903	oadB	G	A
908687	M5005_Spy0920		C	T
948344	M5005_Spy0957		A	C
967436	M5005_Spy0980		A	G
1045871	M5005_Spy1073	dltA	G	A
1058475	M5005_Spy1084		G	A
1062670	M5005_Spy1088	obg	G	A
1272188	M5005_Spy1313		T	C
1484362	M5005_Spy1529	shp	C	A

Accesory genome

The only consistent difference in the accessory genome content between $M1_{DK}$ and the $M1_{global}$ strains it has evolved from is the presence of the speC-containing bacteriophage. The phage is 42kb and contains a total of 54 different genes, including the DNAse mf2, but mostly of unknown function. In the assembly for biosample SAMEA113551919 (DK_iGAS_2022_0442) the bacteriophage is located at positions 556066-898405. The bacteriophage is also found in full in Genbank entry CP060268.1 at positions 548755- 590101 with 98% similarity.
